# Novel Bioengineered Cassava Expressing an Archaeal Starch Degradation System and a Bacterial ADP-Glucose Pyrophosphorylase for Starch Self-Digestibility and Yield Increase

**DOI:** 10.3389/fpls.2018.00192

**Published:** 2018-02-26

**Authors:** Ayalew Ligaba-Osena, Jenna Jones, Emmanuel Donkor, Sanjeev Chandrayan, Farris Pole, Chang-Hao Wu, Claire Vieille, Michael W. W. Adams, Bertrand B. Hankoua

**Affiliations:** ^1^College of Agriculture and Related Sciences, Delaware State University, Dover, DE, United States; ^2^Department of Biochemistry and Molecular Biology, University of Georgia, Athens, GA, United States; ^3^Department of Microbiology and Molecular Genetics, Michigan State University, East Lansing, MI, United States; ^4^Department of Biochemistry and Molecular Biology, Michigan State University, East Lansing, MI, United States

**Keywords:** cassava, hyperthermophilic archaeal, *glgC*, starch self-processing, bioethanol, multigene-expression, starch-hydrolyzing enzymes

## Abstract

To address national and global low-carbon fuel targets, there is great interest in alternative plant species such as cassava (*Manihot esculenta*), which are high-yielding, resilient, and are easily converted to fuels using the existing technology. In this study the genes encoding hyperthermophilic archaeal starch-hydrolyzing enzymes, α-amylase and amylopullulanase from *Pyrococcus furiosus* and glucoamylase from *Sulfolobus solfataricus*, together with the gene encoding a modified ADP-glucose pyrophosphorylase (*glgC*) from *Escherichia coli*, were simultaneously expressed in cassava roots to enhance starch accumulation and its subsequent hydrolysis to sugar. A total of 13 multigene expressing transgenic lines were generated and characterized phenotypically and genotypically. Gene expression analysis using quantitative RT-PCR showed that the microbial genes are expressed in the transgenic roots. Multigene-expressing transgenic lines produced up to 60% more storage root yield than the non-transgenic control, likely due to *glgC* expression. Total protein extracted from the transgenic roots showed up to 10-fold higher starch-degrading activity *in vitro* than the protein extracted from the non-transgenic control. Interestingly, transgenic tubers released threefold more glucose than the non-transgenic control when incubated at 85°C for 21-h without exogenous application of thermostable enzymes, suggesting that the archaeal enzymes produced *in planta* maintain their activity and thermostability.

## Introduction

Global energy consumption is predicted to increase by 48% from 580 quadrillion KJ in 2012 to 860 quadrillion KJ in 2040 (U.S. EIA, 2016). However, given the increasing concerns of environmental pollution and climate change due to production and use of petroleum-based fuels and chemicals (Mei and Rakhmatov, 2014), there is a need to develop alternative and renewable energy sources to meet the demands. Bioethanol as biofuel has been widely produced from sugarcane and corn in Brazil and United States, respectively, (Mei and Rakhmatov, 2014; Lopes et al., 2017). In the 2010/2011 crop season alone, grain ethanol refineries consumed 40% of the total United States corn production (Du and McPhail, 2012). This large diversion of grain to ethanol has led to growing concern over crop-based bioethanol due to its competition with food and feed supplies. Hence, there is a strong interest in second and third generation biofuels (e.g., lignocellulosic biomass and algae) whose technologies are still expensive due to the cost of feedstock production and processing (Carriquiry et al., 2011; Balan, 2014). Therefore, there is a need to identify alternative plant species that can be economically more viable in the near term, are compatible with existing starch-based biofuel technologies, and are high yielding, resilient, and multipurpose (Sanchez and Cardona, 2008; Cooke and Traore, 2010). Starch-rich feedstocks such as cassava (*Manihot esculenta* Crantz) offer great potential to address these needs. Given the ever-increasing demand for food, feed, and energy, as well as continuous changes in our societal demands, there is a need for cost-effective and sustainable starch-processing routes. Recent advances in genetic engineering have enabled the transfer of starch hydrolytic enzymes from the processing tank to starchy feedstock by directly producing enzymes in the biomass (Hebelstrup et al., 2015). This transfer requires the use of thermostable and thermoactive enzymes isolated from hyperthermophiles, which have very low activity at ambient temperatures, and thus do not compromise normal plant growth and development.

Cassava is one of the most important food crops, feeding more than a billion people in about 105 countries in Africa, Asia, and Latin America. It can be grown by resource-poor farmers due to its outstanding adaptation to and productivity on marginal lands (El-Sharkawy, 2004; Chetty et al., 2013). Increasing demands for food, feed, and fuel throughout the tropics has led to a 24% increase in land committed to cassava cultivation since 1990, surpassing the *Solanum* genus potato (Taylor et al., 2012). Cassava is an important source of starch in tropical and subtropical countries, ranking second to corn (De Souza et al., 2016; Karlstrom et al., 2016). According to the Food and Agriculture Organization of the United Nations, about 8.5 million tons of cassava starch and flour were exported globally in 2015 (FAO, 2015), of which Thailand contributed the largest share (7.9 million tons). The demand from ethanol sectors in Asia is driving rapid growth in world cassava production by an estimated 100 million tons (∼35% of total production) since 2000 (FAO, 2013), with at least 780 million liters of cassava ethanol produced annually in China alone (Xuan et al., 2015). Cassava is inherently able to produce very high harvestable yields (>070 tons/ha) of starchy roots in optimum environments, compared to corn (20 tons/ha), sorghum (13 tons/ha), rice (26 tons/ha), sweet potato (65 tons/ha), and wheat (12 tons/ha) (El-Sharkawy, 2004). Moreover, its remarkable tolerance to adverse conditions, such as poor soils, drought, and high temperature (El-Sharkawy and Cock, 1984; Angelov et al., 1993; El-Sharkawy, 1993; Fermont et al., 2009), where most other staple food crops would fail to produce reasonable yields (Jarvis et al., 2012), makes cassava one of the most promising crops in the face of climate change.

Improvement of cassava through traditional breeding has been challenging due to high heterozygosity, low natural fertility, erratic flowering and the lack of agronomically important genes in sexually compatible germplasm (Puonti-Kaerlas et al., 1999; Ihemere et al., 2006; Que et al., 2010; Ceballos et al., 2015; Chavarriaga-Aguirre et al., 2016). Therefore, genetic engineering is a preferred approach for trait improvement. Several laboratories have genetically engineered cassava for agronomically important traits such as vitamin content (Welsch et al., 2010; Li et al., 2015), virus resistance (Vanderschuren et al., 2009, 2012; Beyene et al., 2017), extended shelf-life (Zidenga et al., 2012; Xu et al., 2013), reduced toxic cyanogenic glycoside content (Siritunga and Sayre, 2004; Jorgensen et al., 2005), improved starch quality (Zhao et al., 2011), and increased cold-tolerance (Xu et al., 2014). Likewise, enhanced starch content has been reported in transgenic cassava expressing a mutated *Escherichia coli* ADP-glucose pyrophosphorylase (AGPase; g*lgC*_Gly336Asp_), which is less sensitive to its activator (fructose-1, 6-bisphosphate) and inhibitors (AMP and inorganic phosphate) (Stark et al., 1992; Meyer et al., 1998; Ballicora et al., 2003; Ihemere et al., 2006). AGPase catalyzes the first dedicated and rate-limiting step in starch biosynthesis (Preiss, 1988; Tiessen et al., 2002). Given that cassava is inherently able to produce high yields (El-Sharkawy, 2004), and has the highest annual bioethanol yield compared to other starchy feedstocks such as sweet sorghum, rice, maize, and wheat (Wang, 2002; Ziska et al., 2009), there is a need to develop novel cultivars with increased starch yields and starch self-processing ability for enhanced bioethanol production.

Hyperthermophilic bacteria and archaea growing optimally at temperatures above 80°C and have been a source of thermostable industrial enzymes (Richardson et al., 2002; Egorova and Antranikian, 2005). Complete starch breakdown to glucose requires the synergistic action of thermostable amylases, pullulanases, and glucoamylases (Egorova and Antranikian, 2005). Archaeal amylases (EC 3.2.1.1) have been isolated and characterized from *Pyrococcus furiosus*, *P. woesei*, *Thermococcus profundus*, and *T. hydrothermalis* (Dong et al., 1997a; Jorgensen et al., 1997; Bertoldo and Antranikian, 2001; Mehta and Satyanarayana, 2013). Similarly, pullulanases (EC 3.2.1.41) have been isolated and characterized from the archaea *P. furiosus, P. woesei*, and *Desulfurococcus mucosus* (Rudiger et al., 1995; Dong et al., 1997b; Duffner et al., 2000; Lee et al., 2006; Hii et al., 2012). Glucoamylases (E.C. 3.2.1.3), which convert partially processed starch/dextrin to glucose, have also been isolated from archaea such as *Sulfolobus solfataricus, Picrophilus torridus*, and *Thermoplasma acidophilum* (Bertoldo and Antranikian, 2001; Kim et al., 2004; Kumar and Satyanarayana, 2009) and characterized.

Several laboratories have successfully expressed thermostable bacterial α-amylase/amyllopullulanase in plants, including corn (Lanahan et al., 2006), rice (Chiang et al., 2005; Xu et al., 2008) and sweet potato (Santa-Maria et al., 2011) to induce starch self-processing ability. However, it remains unclear as to whether increased starch self-processing ability could be conferred by heterologous expression of a suite of hyperthermophilic archaeal enzymes *in planta*. Moreover, expressing multigene starch-degrading genes in cassava from a single T-DNA has never been reported before.

In this study, mutated *E. coli glgC_G336D_* as well as the genes encoding three archaeal starch-hydrolyzing enzymes, α-amylase and amylopullulanase from *P. furiosus* and glucoamylase from *S. solfataricus*, were simultaneously expressed in cassava to enhance yield and starch self-processing ability. Transgenic lines were phenotypically and genotypically characterized. Our findings reveal that simultaneous expression of the archaeal and bacterial genes improves starch self-processing ability and storage root yield, respectively. To our knowledge, this is the first report of *in planta* production and activation of multiple archaeal enzymes that led to increased starch hydrolysis directly in the biomass. This approach has a vast potential to improve cassava and other starch-rich feedstock for the production of bioethanol and industrial products.

## Materials and Methods

### Gene Cloning

The modified *P. furiosus* α-amylase gene containing the catalytic and Ca-binding domains (amylase^Δ1-37^ of accession number WP_014835153, designated herein as α-amylase), and amylopullulanase gene (accession number NC_018092, Bridger et al., 2012), and the *S. solfataricus* glucoamylase gene (accession number NC_017274) were amplified from the chromosomal DNA of each archaea using Phusion Hot Start II High-Fidelity DNA Polymerase (Fisher Scientific, Pittsburgh, PA, United States). The amylase sequence starts with the first methionine and lacks 37 residues from the amino-terminus as compared to the sequence reported earlier (Dong et al., 1997a; Jorgensen et al., 1997). The amylase sequence was expressed in *E. coli* strain Rosetta2-DE3-pLyS and the activity of recombinant protein in degrading starch was validated (**Supplementary Methods** and **Supplementary Figure S1A**). The amylopullulanase (NC_018092) used in this study encodes the entire coding region (1355 amino acids). Prior to using this sequence in the multigene assembly its activity, was also validated using a recombinant protein produced in *E. coli* and pullulan as a substrate (**Supplementary Methods** and **Supplementary Figure S1B**). The coding region of *E. coli glgC* (Acc#S58224) was amplified from pO12 plasmid obtained from Dr. Tony Romeo (University of Florida). A 57-amino acid pea chloroplast transit peptide was fused to the N-terminus of *glgC* to target the protein to the amyloplast, a non-pigmented organelle responsible for starch synthesis and storage. Since single amino acid substitution Gly336Asp has been shown to reduce sensitivity of the enzyme to inhibitors and activators (Stark et al., 1992; Meyer et al., 1998; Ihemere et al., 2006), the mutation was introduced by site-directed mutagenesis. The root-specific promoters of the potato class I Patatin and granule-bound starch synthase (GBSS) were amplified from pUC19 and TA vectors, respectively, obtained from Dr. William Belknap (USDA-ARS, Albany, CA, United States). All primers used to amplify promoters or genes used in this study are listed in **Supplementary Table S1**. The sequence of each primer includes 5′ extra nucleotides, a restriction enzyme site, and a sequence specific to the target promoter or gene.

### Generation of Expression Cassettes and Multigene Assembly

**FIGURE 1:**
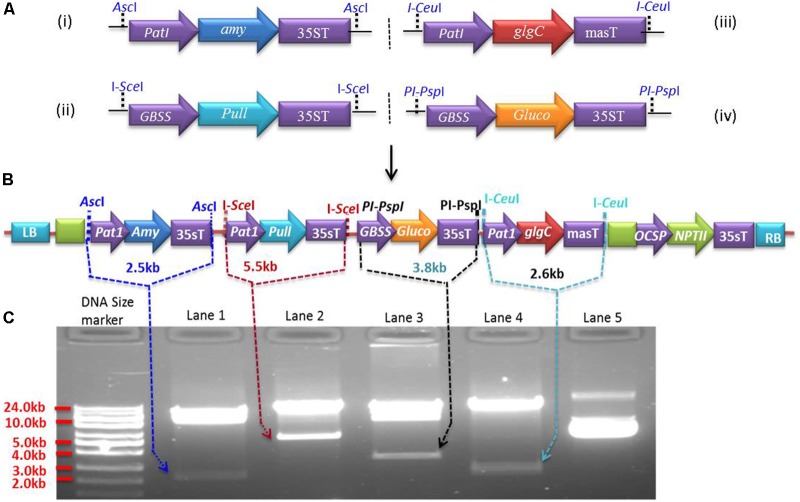
Multigenetic constructs used for cassava transformation. **(A)** Expression cassettes of *Pyrococcus furiosus* α-amylase (pSAT1-*Amy*, **A-i**), *P. furiosus* amylopullulanase (pSAT4-*Pull*, **A-ii**), *Escherichia coli glgC* (pSAT5*-glgC*, **A-iii**), and *Sulfolobus solfataricus* glucoamylase (pSAT6-*Gluco*, **A-iv**) constructed in pSAT modular vectors under the control of Patatin or GBSS promoters. **(B)** The four expression cassettes from **(A)** are assembled into pPZP-RCS2 containing the neomycin phosphotransferase selectable marker (*NPTII*), yielding ∼a 25.61 kb plasmid. **(C)** Validation of the expression cassettes with rare-cutting restriction enzymes (*Asc*I, I-*Sce*I, I-*Ceu*I, and PI-*Psp*I). Lower bands in lanes 1, 2, 3, and 4 represent expression cassettes of *Amy, Pull, Gluco*, and *glgC*, respectively, as indicated by the arrows. Lane 5 is undigested vector containing multigene expression cassettes **(B)**.

Expression cassettes were generated in pSAT modular vectors (pSAT1, pSAT4, pSAT5, and pSAT6) (Chung et al., 2005; Tzfira et al., 2005) under the control of Patatin or GBSS promoters. The Patatin promoter was cloned into pSAT1 and pSAT5, while GBSS was cloned into pSAT4 and pSAT6. Both promoters were cloned upstream of the multicloning sites by replacing the enhanced CaMV promoter (2x35s) in pSAT1, pSAT4, and pSAT6 and the mannopine synthase promoter (mas-P) in pSAT5 (**Figure 1A**). Expression cassettes Patatin-*Amy*-35sT (2.57 kb) for α-amylase, GBSS-*Pull*-35sT (5.68 kb) for amylopullulanase, Patatin-*glgC*-masT (2.72 kb) for *glgC* and GBSS-*Gluco*-35sT (3.48 kb) for glucoamylase were generated in pSAT1 (**Figure 1A-i**), pSAT4 (**Figure 1A-ii**), pSAT5 (**Figure 1A-iii**), and pSAT6 (**Figure 1A-iv**), respectively. The cassettes were then excised from the pSAT vector using *Asc*I and homing endonucleases *I-Sce*I, *I-Ceu*I, and *PI-Psp*I, respectively, and assembled into pPZP-RCS2-ocs-nptII binary vector (8.48 kb) that contains spectinomycin and neomycin phosphotransferase (NPTII, kanamycin) resistance genes for selection in bacterial and plant cells, respectively (Goderis et al., 2002). Intactness of the expression cassettes in the resulting ∼25.6 kb binary vector (**Figure 1B**) was validated using *Asc*I and the homing endonucleases (**Figure 1C**) prior to introduction into *Agrobacterium* strain LBA4404 competent cells. *Agrobacterium* harboring the binary vectors was used to transform cassava friable embryogenic calli (FEC).

### Production of Friable Embryogenic Calli (FEC)

Cassava cultivar TMS60444 obtained from Danforth Plant Science Center was maintained in controlled growth chamber at 27°C, 80% relative humidity and 16-h/8-h day/night cycles. Stem segments with at least one shoot bud were cultured on MS medium (Murashige and Skoog, 1962) with supplements [CBM, 4.43 g/L MS salt with vitamins, 2% (w/v) sucrose, 2 mM CuSO_4_, **Supplementary Table S2**]. Friable embryogenic calli were generated according to Taylor et al. (2001, 2012) The FEC were continuously subcultured on GD (Greshoff and Doy, 1972) 2-50P (**Supplementary Table S2**) media until homogenous clusters were obtained. Homogenous calli were maintained on GD6-50P (supplemented with 6% sucrose) before they were used for transformation or before the calli were used to initiate embryogenic suspension (ES). Embryogenic suspension of the organized calli was initiated and maintained in Schenk and Hildebrandt SH6 (Schenk and Hildebrandt, 1972) modified medium [3.2 g/L SH salt, 6% (w/v) sucrose, 42 μM picloram, **Supplementary Table S2**]. Highly proliferating FEC or ES were used for transformation.

### *Agrobacterium*-Mediated Transformation of Target Tissue

**FIGURE 2:**
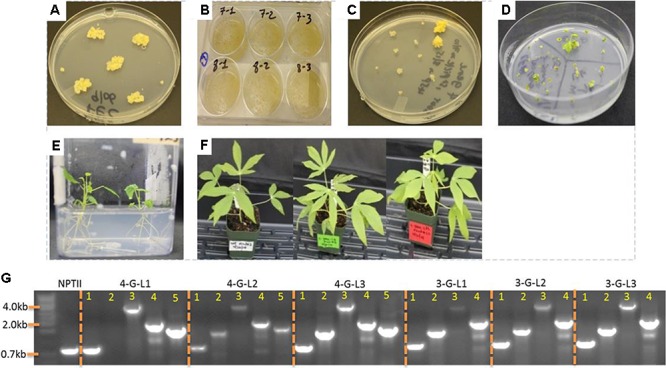
Transformation of cassava friable embryogenic calli (FEC) and generation of transgenic lines. **(A)** FECs were generated as described in the Section “Materials and Methods.” **(B)** Inoculation of FEC with *Agrobacterium* strain LBA4404 containing expression cassettes of starch hydrolyzing enzymes and *glgC* as in **Figure 1**. **(C)** Putative transgenic calli selected under increasing concentrations of paromomycin. **(D)** Shoot regeneration from transgenic lines. **(E)** Rooting of transgenic lines. **(F)** One-month-old non-transformed control and representative transgenic cassava lines. **(G)** Integration of multiple genes into cassava genome as confirmed by PCR using genomic DNA as template. Primers specific to *NPTII, Amy, Pull, Gluco*, and *glgC* were used. When applicable, the bands in lanes 1, 2, 3, 4, and 5 represent *NPTII, Amy, Pull, Gluco*, and *glg*C, respectively.

*Agrobacterium tumefaciens* strain LBA4404 harboring the control binary vector pPZP-RCS2-ocs-nptII and the vectors containing expression cassette of each starch-hydrolyzing enzymes (α-amylase, amylopullulanase, and glucoamylase genes) with or without *glgC* were used for transformation. Single colonies of LBA4404 carrying each construct were cultured in 2 mL LB medium containing appropriate antibiotics for about 10-h while shaking at 220 rpm at 28°C. About 1–1.5 mL of the suspension was used to initiate 25 mL of YM medium containing appropriate antibiotics and 100 μM acetosyringone. The culture was allowed to grow overnight to reach an OD_600_ of 0.5–0.75. The bacteria suspension was prepared according to Taylor et al. (2012). The non-ionic surfactant Pluronic F-68 was added to the bacterial suspension at a concentration of 0.03%. Embryogenic tissues obtained from GD2-50P agar media (**Supplementary Table S1**) or suspension culture was inoculated with the bacterial suspension in 6-well plates (**Figure 2**). At least two independent transformations were done for each construct. The tissues were vacuum-infiltrated for a total of 15 min with two pauses every 5 min, and incubated at room temperature for 30 min. Removal of excess *Agrobacterium* and coculturing was performed according to Taylor et al. (2012). After 5 days of-coculture, the tissues were transferred to Stage I selection medium GD2-50P containing 25 μM paromomycin and 500 mg/L carbenicillin. Putative transgenic tissues were further selected on GD2-50P containing 30 μM (Stage II) and 40 μM (Stage III) paromomycin each for 3 weeks. Transgene insertion was validated by PCR from genomic DNA samples isolated from proliferating putative transgenic calli.

### Maturation and Regeneration of Putative Tissues

After selection under increasing paromomycin concentration (25, 30, and 40 μM), resistant calli were transferred to embryo maturation media (MSN, 4.43 g/L MS salt with vitamins, 2% (w/v) sucrose, 1 mg/L NAA, pH5.8) (Bull et al., 2009) as in **Supplementary Table S1**. Maturing cotyledonary stage embryos were transferred to embryo germination media [CEM, 4.43 g/L MS salt with vitamins, 2% (w/v) sucrose, 0.4 mg/L BAP %, **Supplementary Table S2**]. Germinating shoots were transferred to CBM without antibiotics for rooting.

### Transplanting *in Vitro* Propagated Plants to Soil

Eighteen-day-old uniform micropropagated plantlets (at least nine per construct) were transplanted in Kord 3.0 square pots filled with 230 mL Fafard 51 mix^1^ that has been shown to promote tuber development, and treated with Gnatrol to control fungal gnats (Zidenga et al., 2012). The plants were established in the greenhouse under natural light and fertilized every 3 weeks with NPK (9-45-15) fertilizer. After 4 months when initiation of mini tubers was observed, plants were transferred to 2-L pots and grown for another 3 months before harvesting.

### Quantitative Real-Time RT-PCR

To determine transcript levels of the archaeal genes in cassava tubers, quantitative RT-PCR was performed using an ABI 7500 real-time PCR system and SYBR Green Kit (Applied Biosystems, Grand Island, NY, United States). Total RNA isolated from the flesh (most inner tissue) of tubers using Spectrum Plant Total RNA Kit (Sigma) was treated with DNase I (Invitrogen, Carlsbad, CA, United States) to remove contaminating genomic DNA prior to first-strand complementary DNA (cDNA) synthesis. First-strand cDNA was synthesized using Superscript III (Life Technologies, Grand Island, NY, United States). One microgram of the first-strand cDNA was used for each real-time RT-PCR reaction containing 2xPower SYBR Green Master Mix and 0.15 μM primers in a final volume of 25 μl. Gene-specific sense and antisense primers (**Supplementary Table S3**) were used for the amplification, and cassava α-tubulin (TC30055) was used as an internal control. Relative expression levels were calculated using the ΔΔ*C*_T_ method available on SDS software (Applied Biosystems).

### Total Protein Extraction from Fibrous Roots and Tubers

Total protein from fibrous or tuberous roots was extracted according to Santa-Maria et al. (2011) with minor modifications. The roots or tubers obtained from four independent plants were ground under liquid N_2_, and the ground tissue was homogenized for 10 min using 1.5 mL/g of cold extraction buffer containing 50 mM Tris-HCl (pH 8.0), 0.1 M KCl, 5% glycerol, 1.5% polyvinylpolypyrrolidone, 5% dimethylsulfoxide, 5 mM dithiothreitol, and 1x protease inhibitor cocktail (Sigma–Aldrich, St. Louis, MO, United States). The homogenate was centrifuged at 1,500 × *g* for 10 min. The supernatant was filtered through 250 micron mesh, heated at 80°C for 25 min to eliminate mesophilic enzymes which may interfere with the thermophilic enzyme activity assay, and centrifuged at 4°C for 15 min at 19000 × *g*. For assaying AGPase activity, total protein was extracted from fibrous roots of soil grown plants. Roots (100 mg) were transferred to 2 mL microcentrifuge tubes containing 1 mL extraction buffer containing 100 mM HEPES (pH 7.5), 8 mM MgCl2, 2 mM EDTA, 1 mM DTT, 12.5% (v/v) glycerol and 5% polyvinylpyrrolidone (Kulichikhin et al., 2016). Tissue was homogenized for two 15-s slow-speed periods followed by two 15-s fast-speed blending periods according to Ihemere et al. (2006). The suspension was filtered through miracloth and the extract was centrifuged for 20 min at 21, 000 × *g*. The supernatant was recovered and immediately frozen until it was used for AGPase assay. Total protein was estimated using Coomassie (Bradford) protein assay (Thermo Scientific Pierce, Waltham, MA, United States) using bovine serum albumin as the standard.

### Assay of Enzymatic Activity

Archaeal enzymatic activity was assayed in 1.5 mL of 50 mM phosphate buffer (pH 6.0) containing 15 μg of heat-purified extract and 15 mg pure potato starch (Sigma). For total protein extracted from mini tubers, the activity of enzymes α-amylase, amylopullulanase, and glucoamylase was assayed in reactions containing 1% potato starch and pullulan (Sigma), and 2% maltose (Sigma)/maltotriose (Fisher Scientific), respectively. The reaction was conducted in phosphate buffer at pH 7.0 for starch and pullulan, and pH 5.6 for maltose/maltotriose. The reactions were incubated at 85°C for 30 min and centrifuged at 10,000 × *g* for 2 min. Total glucose in 500 μL of the hydrolysate was determined by the modified 3, 5-dinitrosalicylic acid (DNS) method (Miller, 1959). Increase in absorbance due to the release of glucose was measured at OD_540_. AGPase activity was assayed according to Kulichikhin et al. (2016) based on coupling enzymatic reactions where the product of the initial reaction is used as a substrate for subsequent reactions generating NADPH as the end product. The increase in absorbance due to NADPH was measured at OD_340_ using a control reaction without the enzyme as a reference.

### Assay of Tuber Self-Processing Ability

Saccharification ability of the transgenic tubers was analyzed alongside with the wild-type control. Tubers were harvested and stored at -80°C until used. Frozen tubers were thawed and the bark and peels were removed. The inner starchy tissue (flesh) from same plant was pulled, and ground under liquid N_2_. Tubers obtained from four individual plants were analyzed for each treatment. To determine free sugar content, 1 g of the ground tissue was mixed thoroughly with 2 mL of water. Samples were centrifuged at 10,000 × *g* for 5 min and the supernatant was recovered to determine sugar content. To determine tuber starch self-processing ability, samples were incubated at 85°C, a temperature typically used for starch hydrolysis (Nahampun et al., 2013), for 21-h to activate the thermophilic enzymes. The reaction was cooled to room temperature and the samples were centrifuged for 2 min at 10,000 × *g*. The supernatant was recovered and used to quantify total reducing sugar using the DNS method as mentioned above.

### Statistical Analysis

Experiments were conducted in a complete randomized design in at least three replicates, and experiments were repeated twice. Data were analyzed using one-way ANOVA using the PROG GLM procedure (Westfall et al., 1996). After significant *F*-tests, the Tukey multiple comparison procedure was used to separate the means (*P* < 0.05).

## Results

### Enzyme Cocktails from Fibrous Roots of Transgenic Cassava Hydrolyze Starch *in Vitro*

Starch conversion to glucose requires high temperature treatment and the action of thermostable glycosyl hydrolases that are commercially produced from thermophilic microorganisms. To study whether the archaeal enzymes are produced in the roots, the genes were targeted to the roots using root-specific promoters. A total of 11 lines expressing the three starch-degrading enzymes and the mutated bacterial AGPase (designated as 4-G-lines), two lines expressing the starch-degrading enzymes (3-G-lines), and five empty vector control (NPTII) lines were obtained. During *in vitro* growth and 3 weeks after transplanting to soil, the performance of the transgenic lines expressing multiple genes was not different from the non-transgenic control plants (**Figure 2F**). These results may suggest that expression of multiple hyperthermophilic archaeal enzymes does not interfere with normal plant growth and development due to these enzymes’ extremely low catalytic activities at ambient temperature.

Initial characterization of the transgenic lines was based on starch-degrading activity of enzymes extracted from fibrous roots. Plants were propagated *in vitro* on agar media and allowed to grow for at least 1 month until sufficient fibrous roots were obtained for protein extraction. Total protein from the roots was extracted and purified by heating, and used for saccharification of purified potato starch *in vitro*. The amount of glucose produced due to starch-hydrolyzing activity of the recombinant enzyme cocktails was determined using the DNS colorimetric assay.

**FIGURE 3:**
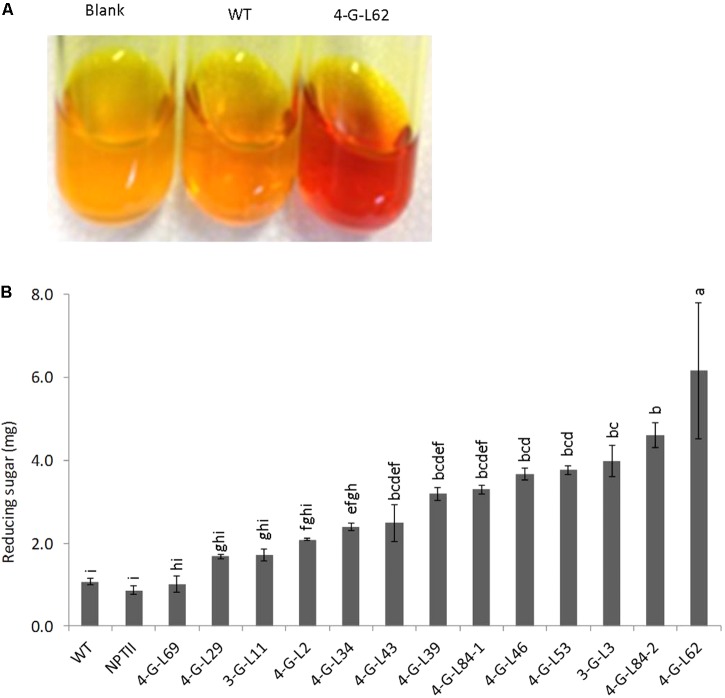
Starch-degradation assay as determined by the DNS method. **(A)** Increase in color intensity of the DNS reagent with enzyme cocktail from wild-type (WT, non-transgenic), empty vector (NPTII) and transgenic lines (3-G, expressing the starch-degrading enzymes; 4-G, expressing the starch-degrading enzymes and AGPase). **(B)** Hydrolysis of purified potato starch by enzyme cocktail extracted from fibrous roots of control and transgenic cassava lines. The resulting reducing sugar/glucose was quantified by DNS colorimetric assay method using a maltose solution as the standard. Color intensity of DNS reagent was measured at 540 nm. Bars represent mean ± standard errors (SE) based on three biological replicates. Bars bearing the same letter are not statistically different (*p* < 0.05).

As shown in **Figure 3**, statistically significant difference (*p* < 0.0001) was observed between the starch hydrolyzing activities of heat-purified (80°C for 25 min) protein extracts from fibrous roots of transgenic and that of control lines (WT and NPTII). Protein extracts from the majority of transgenic lines released more glucose from starch than those from wild-type and empty vector control lines. The enzyme cocktail from 4-G-L62 coexpressing the three starch-hydrolyzing enzymes (α-amylase, amylopullulanase, and glucoamylase) and *E. coli* AGPase showed the highest starch-degrading activity, releasing about sixfold more glucose than the wild-type control. Likewise, 3-G-L3, which expresses only the starch-hydrolyzing enzymes, released about fourfold more glucose than the wild-type control. This finding suggests that hyperthermophilic archaeal enzymes maintain their activity and thermostability when heterologously expressed in cassava roots.

### Characterization of Greenhouse- Established Transgenic Plants

To further characterize the multigene expressing lines, *in vitro* propagated transgenic and non-transgenic control plants were transplanted to soil according to Zidenga et al. (2012) and transferred to the greenhouse. Plants were grown under natural light from April to December.

**FIGURE 4:**
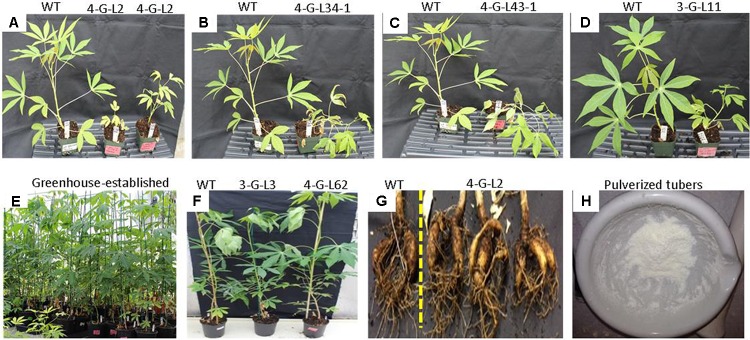
Characterization of transgenic lines grown in the greenhouse. **(A–D)** Transgenic lines exhibiting abnormal growth characteristics as compared to the non-transgenic control. **(E)** Greenhouse-established 6-month old plants. **(F)** Comparison of WT, 3-G-L3, and 4-G-L62 biomass. **(G)** Tubers harvested from 7-month-old WT and 4-G-L62. **(H)** Tubers pulverized under liquid N_2_ for protein or RNA extraction, or analysis of starch self-processing ability.

While the transgenic lines developed normal shoots and fibrous roots on MS media in controled growth chambers and during the first 3 weeks after transplanting to soil (**Figure 2F**), we observed variations after greenhouse establishment in a later stage for most transgenic lines (**Figure 4** and **Table 1**). The growth of 4-G-lines (4-G-L2 and 4-G-L53) and 3-G-L11 was severely stunted while 4-G-L34 and 4-G-L43 produced creeping shoots with weak stems (**Figures 4A–D**). This phenotypic alteration could be due to somaclonal variation as previously reported in cassava (Raemakers et al., 2001; Taylor et al., 2012). Since the FEC used in this study was about 4-months old, it is likely that the observed growth abnormality of the plants could be due to the age of the FEC. This is consistent with Taylor et al. (2012) who suggested that transformation of FEC at an optimum stage (about 9 weeks after induction), and timely subculturing of transgenic tissues could reduce the formation of abnormal plants. The observed growth abnormality could be due to genetic or epigenetic variation (Machczynska et al., 2015).

**Table 1:** Agronomic traits of wild-type and transgenic cassava lines.

Line	Plant height (g)	Stem fresh weight (g)	Tubernumber	Tuber fresh weight (g)
WT	143 ± 2^cb^	61 ± 1^ab^	2.0 ± 0.0^b^	20 ± 3.1^a^
3-G-L3	138 ± 5^C^	52 ± 2^b^	1.3 ± 0.3^b^	4.0 ± 1.4^b^
4-G-L29	155 ± 3^b^	63 ± 4^ab^	1.3 ± 0.3^b^	20 ± 2.0^a^
4-G-L62	171 ± 6^a^	74 ± 3^a^	3.7 ± 0.3^a^	32 ± 7.5^a^

*The agronomic parameters (plant height, stem fresh weight, number of tubers, and tuber total fresh weight) were measured on 7-month-old plants. Values represent means ± SE based on three replicates. Means followed by same letter are not statistically different (p < 0.05).*

**FIGURE 5:**
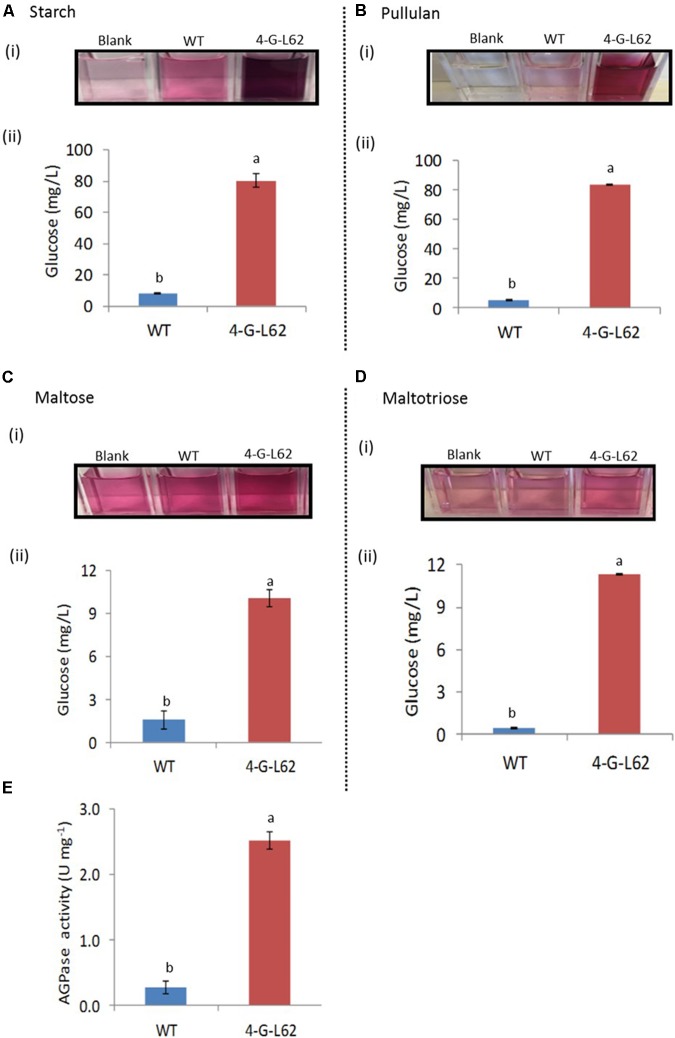
Carbohydrate-hydrolyzing activity of enzyme cocktails extracted from tubers of WT or transgenic (4-G-L62) cassava as determined by the glucose oxidase–peroxidase assay. Degradation of starch **(A)**, pullulan **(B)**, maltose **(C)** and maltotriose **(D)** by heat-purified enzyme cocktail from WT (blue bar) and 4-G-L62 (red bar). (i) Increase in color intensity due to the release of glucose from each carbohydrate. (ii) Amount of glucose released from each polysaccharide as determined from a change in color intensity measured at 540 nm using glucose as a standard. **(E)** AGPase activity assay in roots of WT (blue bar) and 4-G-L62 (red bar) based on an increase in absorbance at OD_340_ due to NADPH formation generation. Bars represent mean ± SE based on four replicates. Bars bearing the same letter are not statistically different (*p* < 0.05).

Initiation of tuberous root was observed after 4-month of growth in the soil. The frequency and time of tuber initiation varied among the greenhouse-established lines. The plants were allowed to grow for 7 months. Line 3-G-L3 (expressing only the archaeal genes) produced smaller shoot and tuber biomass than both the wild-type and 4-G-lines after 7 months (**Table 1**). Line 4-G-L62 whose root enzyme extract produced the highest amount of glucose (**Figure 3**) also produced more shoot and tuber biomass (**Figures 4F,G** and **Table 1**) than the control, 3-G-L3, and 4-G-L29. Interestingly, the average number of tubers per plant, which is positively correlated with yield (Edet et al., 2015), was also significantly higher in 4-G-L62 than the other transgenic lines and in the non-transformed control (**Figure 4G** and **Table 1**). This yield increase in 4-G-L62 is likely due to coexpression of modified *glgC* (G336D) (**Table 2**) in line with increased AGPase activity in 4-G-L62 (**Figure 5E**). However, the increase in storage root observed in transgenic TMS60444 in this study is lower than what was reported in *glgC* expressing TMS 71173 (Ihemere et al., 2006), which could be due to genotypic difference or growth conditions, for example, facilities for controlling light, temperature and humidity. Cassava growth and photosynthetic assimilate partitioning are largely influenced by environmental factors such as temperature and photoperiod (El-Sharkawy, 1993; Rao et al., 2016).

**Table 2:** Expression of archaeal (*Amy*, *Pull*, and *Gluco*) and bacterial (*glgC*) genes in tubers of wild-type (WT) and multigene-expressing lines as determined by qPCR.

Line	Amy	Pull	Gluco	glgc
WT	1.0 ± 0	1.0 ± 0	1.0 ± 0	1.0 ± 0
4-G-L29	1.0 ± 0	5.0 ± 2.7	13.0 ± 7.4	2.0 ± 0.2
4-G-L62	1137 ± 48	924 ± 758	6.0 ± 2.5	3245 ± 318

*Amy, α-amylase; Pull, amylopullulanase; Gluco, glucoamylase. Values represent means ± SE of fold change in expression as compared to the non-transformed control based on four biological replicates*.

### Archaeal and Bacterial Genes Are Expressed in Transgenic Cassava Tubers

Given that the enzyme cocktail extracted from fibrous roots of transgenic lines significantly increased saccharification of starch *in vitro* as compared to the non-transgenic control (**Figure 3**), we studied transcript accumulation in the tubers to confirm expression of the archaeal and bacterial genes in transgenic storage roots. Total RNA was isolated from the inner part of the tuber (flesh, the part of the storage root used for starch extraction) of WT, 4-G-L29, and 4-G-L62. Transcript accumulation was quantified by quantitative RT-PCR.

As shown in **Table 2**, a significant increase in α-amylase, amylopullulanase, glucoamylase, and *glgC* transcript levels was observed in tubers of 4-G-L62 as compared to both WT and 4-G-L29. Amylopullulanase and glucoamylase transcript levels were also higher in 4-G-L29 than in the control, while no increase in the transcripts of α-amylase, and only a slight increase in that of *glgC* were observed in 4-G-L29. The observed increase in transcript accumulation of the archaeal genes is consistent with the higher starch degradation activity of the enzyme cocktail extracted from fibrous roots of transgenic lines as compared to the control (**Figure 3**). This correlation is more evident for amylase and amylopullulanase since expression of the two enzymes was higher in line 4-G-L62, which released the highest amount of glucose. Likewise, increased *glgC* transcript level in 4-G-L62 correlates well with enhanced tuber and overall biomass yield (**Table 1** and **Figure 4G**).

### A Cocktail of Enzymes from Transgenic Tubers Degrade Carbohydrates *in Vitro*

To determine the activity of the individual archaeal enzymes in the root-derived enzyme cocktail, we analyzed the activity of the enzyme cocktails extracted from tubers of 4-G-L62 and the WT on purified starch, pullulan, and maltotriose/maltose, chosen as substrates for amylase, amylopullulanase, and glucoamylase, respectively.

The enzyme cocktail extracted from 4-G-L62 showed increased saccharification of all the tested carbohydrates (**Figure 5**). The amount of glucose released from starch by the 4-G-L62 enzyme cocktail was at least 10-fold higher than the enzyme cocktail from WT control (80 mg/L vs. 8 mg/L) (**Figure 5A**). Likewise, purified proteins from 4-G-L62 released significantly more glucose from pullulan, which is a suitable substrate for amylopullulanase, than the proteins extracted from the WT. The activity of enzymes from 4-G-L62 on maltose and maltotriose (substrates of glucoamylase) was also higher than the WT (**Figure 5B**), suggesting that the archaeal enzymes are expressed in their active forms. The basal saccharification level observed for WT as compared to the control without a substrate could be due to substrate hydrolysis by heat.

### Production of Hyperthermophilic Starch-Degrading Enzymes in Transgenic Tubers Enhance Starch Self-Processing Ability

Given that the primary goal of this study was to develop cassava with starch self-processing ability by directly producing thermostable starch-hydrolyzing enzymes in the tuber, we compared the self-processing ability of tubers from 4-G-lines to that of the non-transformed control. Starch self-processing ability was determined based on total reducing sugar content before and after heat incubation.

**FIGURE 6:**
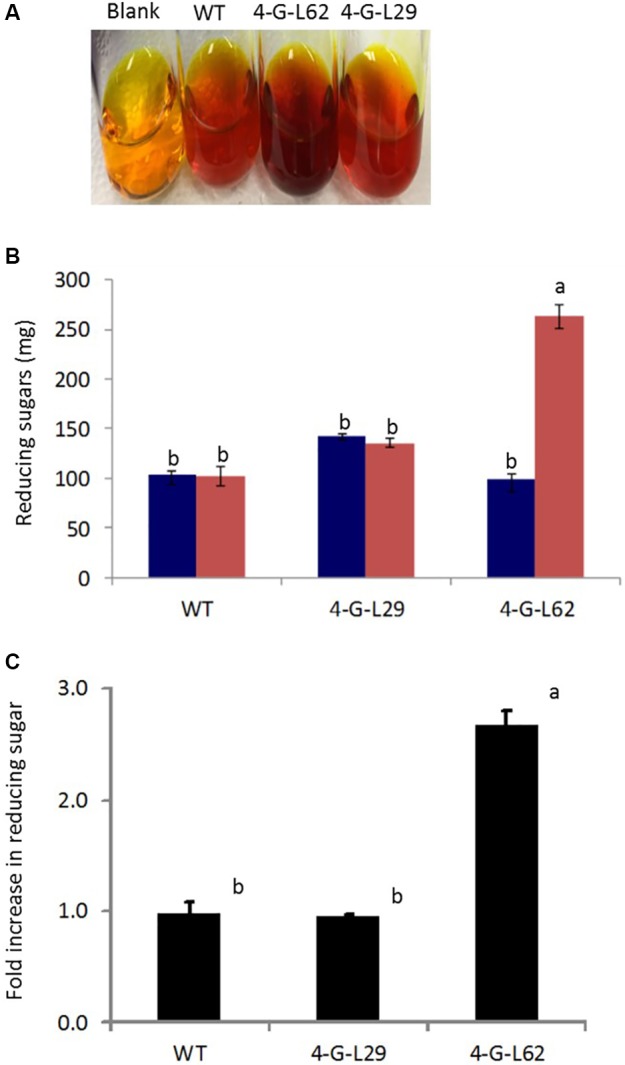
Starch self-processing ability of WT and transgenic (4-G-L29 and 4-G-L62) tubers. **(A)** Increase in color intensity due to the release of glucose from storage roots after incubation at 85°C for 21-h. **(B)** Reducing sugar content determined from a change in color intensity of DNS reagent before (blue bars) and after (red bars) incubation at 85°C measured at 540 nm using maltose as the standard. **(C)** Fold increase in reducing sugar after 21-h incubation at 85°C as compared to initial sugar content. Bars represent mean ± SE based on four biological replicates. Bars bearing the same letter are not statistically different (*p* < 0.05).

As shown in **Figure 6**, initial total reducing sugar content in the tubers was not significantly different between the transgenic lines and the wild-type control, suggesting that the thermophilic enzymes do not degrade starch in the intact tubers. After incubation of the tuber biomass for 21-h at 85°C, multigene expressing 4-G-L62 released significantly more reducing sugar than the WT, while 4-G-L29 was not different from the WT (**Figures 6A,B**). Lack of self-processing ability in 4-G-L29 could be due to a low level of enzyme accumulation, consistent with a low level of gene expression as compared to 4-G-L62. In particular, the α-amylase transcript did not accumulate in the tubers of 4-G-L29 beyond the background level (**Table 2**). The reducing sugar yield was three times higher in 4-G-L62 than in 4-G-L29 and the WT (**Figures 6A,C**). These findings establish that accumulation of starch hydrolyzing enzymes induce tuber self-processing ability.

## Discussion

### Recombinant Archaeal Enzymes Maintain Their Catalytic Activity and Thermostability

In this study, expression of a suite of archaeal starch-degrading enzymes was targeted to the roots of cassava. To understand whether the enzymes are accumulated in their active forms, total protein was extracted from storage roots was used for saccharification of carbohydrates including starch, pullulan, maltose/maltotriose as substrates for amylase, amylopullulanase and glucoamylase, respectively.

The findings revealed that recombinant enzyme cocktails extracted from storage roots of the transgenic line showed significantly more carbohydrate-hydrolyzing activity, as compared to the cocktail extracted from the non-transgenic control (**Figures 3**, **5**). This increase in carbohydrate saccharification establishes that the archaeal enzymes maintain their activity and thermostability when produced in cassava roots. The amount of glucose released from starch and pullulan by the 4-G-L62 enzyme cocktail was over 10-fold more than the enzyme cocktail from WT. Higher activity of the cocktail on starch and pullulan is likely due to higher expression of α-amylase and amylopullulanase (**Table 2**) as compared to glucoamylase, since amylopullulanase is a bifunctional enzyme possessing both pullulanase and amylase activities (Kim et al., 1993; Chiang et al., 2005). The amount of glucose released from both maltose and maltotriose was much lower than those released from starch or pullulan, which is likely due to low level of gene expression. Taken together, our findings establish that active forms of a suite of thermophilic carbohydrate-degrading enzymes can be produced *in planta*. Note, though, that this conclusion is based on the cumulative activity of the enzyme mix, and that the specific activity of each recombinant enzyme needs to be determined separately in the future. Furthermore, since the assays were performed at 85°C, a temperature typically used for starch hydrolysis, assay conditions such as temperature, pH, buffer, and ions need to be optimized for the three enzymes in the cocktail.

The observed increase in saccharification of several carbohydrates due to targeted accumulation of the archaeal enzymes in the tubers suggests a potential of the tubers for commercial-scale production of high-value enzymes because tubers have specialized molecular environment for product stability as previously reported in potato (Sparrow et al., 2007). Targeting of the proteins to the apoplast may further enhance enzyme production and improve saccharification efficiency. For example, targeting expression of hydroxynitrile lyase to the apoplast under the control of Patatin promoter has been shown to enhance protein accumulation by threefold in cassava (Narayanan et al., 2011). Likewise, accumulation of the enzymes may also be targeted to the endoplasmic reticulum (ER), which has several advantages including being a suitable environment for correct folding and disulfide bridge formation by the ER chaperones and enzymes (Schillberg et al., 1999; Wilkinson et al., 2005), and low levels of proteolytic activity in the ER lumen as compared to the cytosol.

### Enhanced Self-Processing Ability of Transgenic Tubers

To validate starch hydrolyzing activity of the recombinant hyperthermophilic enzymes directly in the tubers, the tubers were pulverized, incubated at 85°C overnight to activate the enzymes, and the total reducing sugar yield in the hydrolysate was quantified. The result shows that transgenic tubers release three-times more reducing sugars as compared to the wild-type control, suggesting that the recombinant enzymes can hydrolyze starch *in situ*. A number of thermostable starch-hydrolyzing genes from bacteria have been expressed in transgenic plants. For example, coexpression of *Bacillus stearothermophilus* bifunctional α-amylase and glucose isomerase in potato tuber (*Solanum tuberosum*) (Beaujean et al., 2000), *B. stearothermophilus*α-amylase in transgenic rice seeds (Xu et al., 2008), *Thermotoga maritima* α-amylase in sweet potato (*Ipomoea batatas*) (Santa-Maria et al., 2011), and *T. thermohydrosulfuricus* truncated amylopullulanase in corn (Nahampun et al., 2013) induced starch saccharification at high temperatures. Interestingly, in all these studies the transgenes have not been shown to affect plant development and metabolism, and harvestable yield. However, cases in which thermostable starch-degrading enzymes modulated plant composition have also been reported, for example, increasing the expression level of thermostable *Thermoanaerobacter ethanolicus* amylopullulanase in rice modified the starch composition by reducing the amylose content (Chiang et al., 2005).

Recombinant hyperthermophilic archaeal glycosyl hydrolases have been produced in bacteria and their carbohydrate degrading activity has been established (Dong et al., 1997a,b; Kim et al., 2004; Lee et al., 2006). Only a few archaeal proteins have been successfully expressed in planta, though, primarily for studying the feasibility of recombinant protein production (Montalvo-Rodriguez et al., 2000; Conley et al., 2011; Klose et al., 2012) or stress tolerance (Im et al., 2009). To our knowledge, this study reports for the first time on heterologous expression of archaeal genes in plants for an *in situ* activation to enhance starch self-processing ability. This novel approach should lead to a sustainable route for starch conversion to sugars for biofuels and other industrial products. Given that cassava is the highest yielding starchy feedstock (Ziska et al., 2009; El-Sharkawy, 2012), and the most resilient crop to environmental variability (El-Sharkawy, 2012; Jarvis et al., 2012), transgenic cassava with improved traits will attract tremendous commercial interest.

### Potential Applications of This Study

In this study we expressed multiple transgenes in cassava tubers to simultaneously induce starch autohydrolysis and increase starch yield. Trait improvement through traditional breeding is challenging in crops such as cassava, sugarcane (*Saccharum* spp.), and the biofuel grass miscanthus (*Miscanthus* spp.) (McGarry et al., 2017). The vector system used in this study offers a platform for multiple gene insertion into these crops to engineer traits that require a suite of enzymes, for example, polysaccharide conversion to sugars for biofuels or other industrial purposes (as in this study), disease resistance (Tricoli et al., 1995; Beyene et al., 2017), pest resistance (Santamaria et al., 2012), enhanced vitamin content (Ye et al., 2000; Lu et al., 2013; Pons et al., 2014), molecular farming of industrial and pharmaceutical products (De Martinis et al., 2016), and simultaneous improvement of two or more traits.

Studies have shown that cassava-derived bioethanol has economic advantages over other starchy feedstocks including corn (Dai et al., 2006; Ziska et al., 2009). The annual bioethanol yield of cassava is three-times that of corn (Wang, 2002; Ziska et al., 2009). Therefore, introducing starch self-processing trait in cassava will further increase its demand as bioethanol feedstock, and will have great economic and environmental benefits. Starch processing to sugar for bioethanol production or in food processing industries is a multistep process that requires the application of commercial enzymes such as α-amylase and glucoamylase. Considering the cost of large-scale enzyme production and applications to bioprocessing, the current approach whereby starch-degrading enzymes are directly produced and accumulated in the plant starchy biomass, and are heat-activated *in situ* to break down starch, would tremendously cut the cost of starchy feedstock processing. This technology has the potential to attract commercial interest, as observed for the genetically modified corn developed by Syngenta (Enogen) that expresses microbial α-amylase for ethanol production (Johnson et al., 2009). Furthermore, the current approach reduces energy/labor cost and increases process efficiency in many ways; (1) the archaeal hyperthermophilic enzymes with temperature optima above 90°C (Dong et al., 1997a,b; Jorgensen et al., 1997; Kim et al., 2004) will circumvent the need for the expensive cooling step normally required before saccharification step in the case of fungal glucoamylases with lower temperature optima (50–70°C) (Norouzian et al., 2006), (2) transgenic storage roots may be processed directly and require less water for grinding, omitting further processing such as drying which requires space, and is labor intensive, (3) provided that the recombinant enzymes are accumulated at higher levels, the transgenic biomass may be blended with non-transgenic biomass to reduce enzyme cost and the need to allocate more land for genetically engineered plants. Moreover, glucose produced by this potentially cheap route can serve as a feedstock for a plethora of industrial products. A number of products including succinic acid, itaconic acid, adipic acid, 3-hydroxypropanoic acid/aldehyde, isoprene/farmesene, glutamic acid and aspartic acid can be produced from glucose through biological fermentation (Sanchez et al., 2005; de Jong et al., 2010). Likewise, glucose can be chemically transformed to useful products such as sorbitol, furfural, glucaric acid, hydroxymethyl furfural and levulinic acid (Kusserow et al., 2003; de Jong et al., 2010).

## Conclusion

In this study, we report on the production of a suite of hyperthermophilic archaeal enzymes that lead to enhanced starch self-processing ability directly in the transgenic biomass. However, to realize the full potential of this technology, recombinant enzyme production should be enhanced, for example, by codon-optimizing the native gene sequences, by using stronger promoters, translation enhancers, and/or by targeting the proteins to cellular compartments, such as apoplast and ER. All of these approaches have been shown to increase protein expression and storage levels elsewhere. To compensate for any potential harvestable yield penalty due to expression of multiple genes, there may be a need to co-express genes that enhance biomass/starch yield. Furthermore, future research will characterize the transgenic lines under well-controlled growth conditions, and establish optimum reaction conditions for the enzyme cocktail, including processing media, temperature, pH and cofactor additions. Moreover, the fermentability of the saccharification product by yeast needs to be investigated. Overall, this study reports on a novel approach for sustainable and inexpensive route to produce fermentable sugars from cassava, which can easily be transferred to other low-input tuber/root crops such as sweet potato, *Solanum* genus potato, and other tropical root crops including yam, taro, and arrowroot.

## Author Contributions

BH, AL-O, CV, and MA conceived the study and secured federal grant to support research activities. AL-O, MA, FP, CV, and BH designed all experiments. AL-O, BH, FP, JJ, SC, C-HW, and ED conducted experiments and collected data. BH, SC, FP, and C-HW cloned the starch hydrolyzing archaeal genes from *Pyrococcus furiosus*. AL-O cloned glucoamylase from *Sulfolobus solfataricus* and *E. coli glgC*, generated all the transgenic cassava plants, performed all biochemical characterization of these lines, and wrote the manuscript. AL-O and BH analyzed the data, designed and constructed and characterized all the expression vectors utilized to recover transgenic cassava lines. AL-O, JJ, and ED performed expression studies of *Pyrococcus furiosus* amylopullulanase and *α*-amylase in *E. coli*. CV, BH, MA, SC, FP, and C-HW edited the manuscript. All authors read and approved the manuscript.

## Conflict of Interest Statement

The authors declare that the research was conducted in the absence of any commercial or financial relationships that could be construed as a potential conflict of interest.

## Footnotes

1 https://www.hummert.com/

## Supplementary Material

FIGURE S1 Validation of enzyme activity of recombinant *Pyrococcus furiosus* α-amylase and amylopullulanase. **(A)** The release of glucose from starch by α-amylase. **(B)** The release of glucose from pullulan by amylopullulanse. Values represent means ± SE based on three replicates.

TABLE S1 Primers used for cloning root specific promoters Patatin I and GBSS, archaeal starch hydrolyzing enzymes and *E. coli* Glgc into pSAT modular vectors for subsequent assembly into pPZP-NPTII binary vector. The sequence of each primer includes 5′ extra nucleotides (italicized), restriction enzyme site (upper case letters and underlined) and a sequence specific to the target promoter or gene (lower case non-italic).

TABLE S2 Types of media and composition used for cassava tissue culture. MS (MS with vitamins; Murashige and Skoog, 1962), GD (Greshoff and Doy, 1972) composition is based on phytotechnology laboratories (https://phytotechlab.com/), SH (Schenk and Hildebrandt, 1972), BAP (6-Benzylaminopurine), NAA (Naphthalene acetic acid). Media were solidified by phytagel (0.22%) for CBM and Noble Agar (0.8%) for all other media. pH (5.8).

TABLE S3 Primers used for qPCR to study expression of archaeal starch hydrolyzing enzymes and *E. coli glgc* from control and transgenic cassava tubers. Cassava α-tubulin gene (TC3055) was used as an internal control.
