# Supplementary figures and images for: Novel Bioengineered Cassava Expressing an Archaeal Starch Degradation System and a Bacterial ADP-Glucose Pyrophosphorylase for Starch Self-Digestibility and Yield Increase
Source: Front Plant Sci. 2018 Feb 26;9:192. doi: 10.3389/fpls.2018.00192 (PMC5836596; doi:10.3389/fpls.2018.00192)

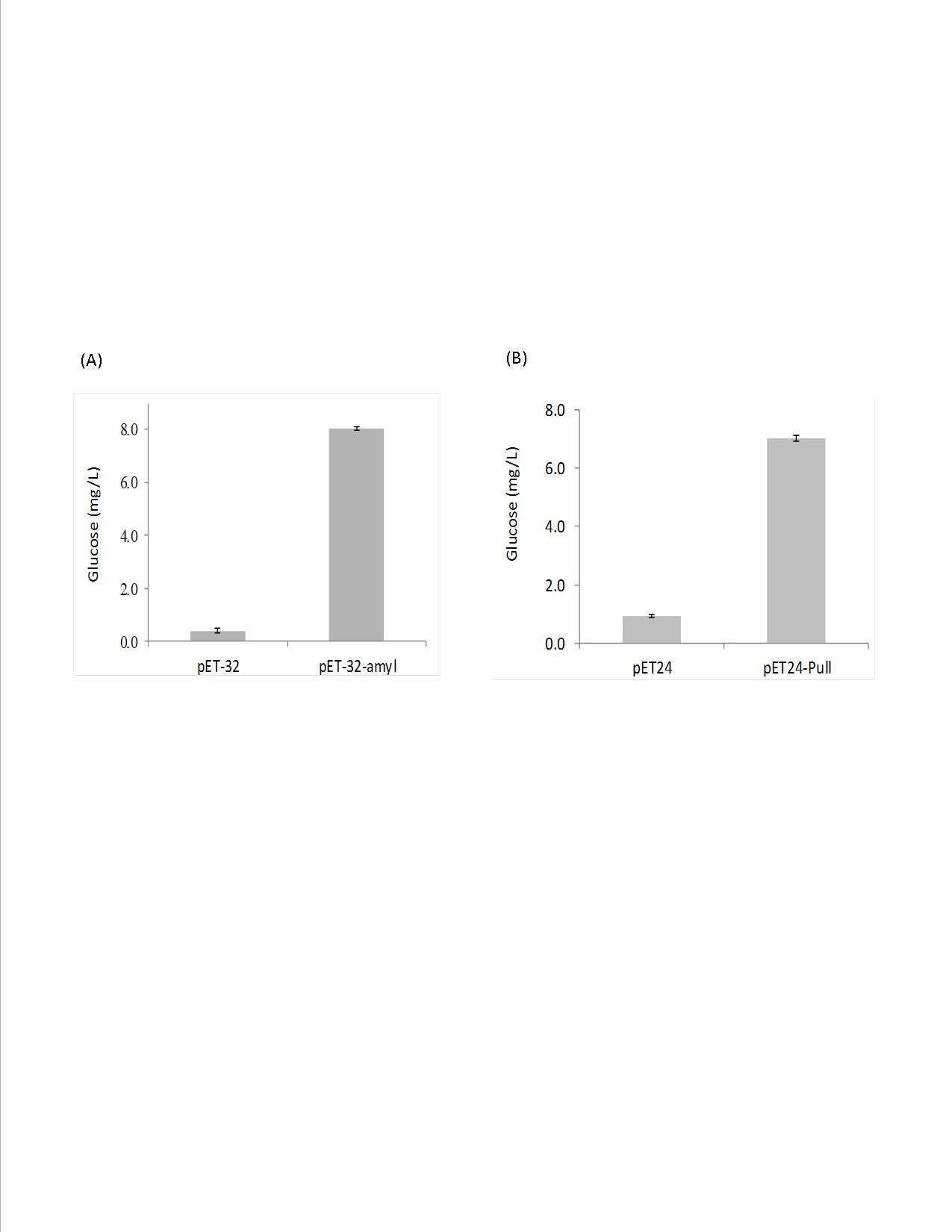

Supplement: FIGURE S1 — Validation of enzyme activity of recombinant Pyrococcus furiosus α-amylase and amylopullulanase. (A) The release of glucose from starch by α-amylase. (B) The release of glucose from pullulan by amylopullulanse. Values represent means ± SE based on three replicates. [file Image_1.JPEG]
