# Supplementary material for: Novel Bioengineered Cassava Expressing an Archaeal Starch Degradation System and a Bacterial ADP-Glucose Pyrophosphorylase for Starch Self-Digestibility and Yield Increase
Source: Front Plant Sci. 2018 Feb 26;9:192. doi: 10.3389/fpls.2018.00192 (PMC5836596; doi:10.3389/fpls.2018.00192)
Supplement: TABLE S1 — Primers used for cloning root specific promoters Patatin I and GBSS, archaeal starch hydrolyzing enzymes and E. coli Glgc into pSAT modular vectors for subsequent assembly into pPZP-NPTII binary vector. The sequence of each primer includes 5′ extra nucleotides (italicized), restriction enzyme site (upper case letters and underlined) and a sequence specific to the target promoter or gene (lower case non-italic). [file Table_1.PDF]

Table S1. Primers used for cloning root specific promoters Patatin I and GBSS, archaeal starch hydrolyzing enzymes and *E.coli* Glgc into pSAT modular vectors for subsequent assembly into pPZP-NPTII binary vector. The sequence of each primer includes 5' extra nucleotides (italicized), restriction enzyme site (upper case letters and underlined) and a sequence specific to the target promoter or gene (lower case non-italic).

Promoter/Gene	Sense primer	Antisense primer
Patatin I	*aaggaaagga*ACCGGTttgttagttaatgcgtattagttttagcgacg	*aaggaaagga*CCATGGgaattcgagctcggtacccggggcaaatgttc
GBSS	*aaggaaagga*CCATGGactatagggcgaattgggccctctagatgcatg	*aaggaaagga*CCATGGatgagagtttcctccataggaaatggcagaatgc
P. furiosus α-amylase	*aaggaaagga*AGATCTatgcaagcattctattgggatgttccagggggag	*gaaagga*AAGCTTcttgtcatcatcgtccttgtagtccccaacaccacaataactc
P. furiosus amylopullulanase	*aaggaaagga*CCGCGGatgagtaggaagctttctctcctcttagtatttc	*aagga*CCCGGGagcgtaatctggaacatcgtatgggtagctccttcttcttaaaac
S. solfataricus glucoamylase	*aaggaaagga*GTCGACatgagagtttcctccataggaaatggcagaatgc	*aagga*CCGCGGcaggtcttcttcagagatcagtttctgttctatatggtttaagagc
E. coli glgc	*aaggaaagga*CTCGAGatggcttctatgatatcctcttccgctgtgacaac	*aagga*CCCGGGgtggtgatgatgatgatgtcgctcctgtttatgccctaac

Table S2. Types of media and composition used for cassava tissue culture. MS (MS with vitamins; Murashige and Skoog 1962), GD (Greshoff and Doy 1972) composition is based on phytotechnology laboratories (https://phytotechlab.com/), SH (Schenk and Hildebrandt, 1972), BAP (6-Benzylaminopurine), NAA (Naphthalene acetic acid). Media were solidified by phytagel (0.22%) for CBM and Noble Agar (0.8%) for all other media. pH (5.8).

Media	Composition	purpose	Reference
CBM	4.43g/L MS, 2% (w/v) sucrose, 2 mM $CuSO_4$	Rooting and *in vitro* propagation	Bull et al. 2009
MS2-P50	4.43g/L MS, 2% (w/v) sucrose, 50 μM picloram, 2 mM $CuSO_4$	initiation of embryogenic calli	Bull et al. 2009
GD2-50P	GD, 2% (w/v) sucrose, 2 mM $CuSO_4$, 50 μM picloram	initiation of FEC	Taylor et al. 2001
GD6-50P	GD, 6% (w/v) sucrose, 2 mM $CuSO_4$, 50 μM picloram	Proliferation and maintenance of FEC	Taylor et al. 2001
MSN	4.43g/L MS, 2% (w/v) sucrose, 1 mg/L NAA	regeneration of transgenic embryos	Bull et al. 2009
CEM	4.43g/L MS, 2% (w/v) sucrose, 0.4mg/L BAP	Shoot induction	Bull et al. 2009
SH6	3.2g/L SH, 6% (w/v) sucrose, 42 μM picloram	Initiation and proliferation	Taylor et al. 2001

Table S3. Primers used for qPCR to study expression of archaeal starch hydrolyzing enzymes and *E.coli glgc* from control and transgenic cassava tubers. Cassava α-tubulin gene (TC3055) was used as an internal control.

Gene	Sense primer	Antisense primer
P. furiosus α-amylase	tgttaagggctatggagcttgg	ccactctcatatgcccagctta
P. furiosus amylopullulanase	agggatagcgttgggttaatga	gcttacctgagcctctccaatc
S. solfataricus glucoamylase	agacggcattagaagtatgggc	gttttggcaatccttcttccat
E. coli glgc	agaactccagccacgactttg	tacccacatcgcgccagtac
Manihot esculenta α-tubulin	caagtgcgatcctcgacatg	gataccgcacttgaacccag
