# Supplementary material for: Novel Bioengineered Cassava Expressing an Archaeal Starch Degradation System and a Bacterial ADP-Glucose Pyrophosphorylase for Starch Self-Digestibility and Yield Increase
Source: Front Plant Sci. 2018 Feb 26;9:192. doi: 10.3389/fpls.2018.00192 (PMC5836596; doi:10.3389/fpls.2018.00192)
Supplement: Supplementary file 3 [file Presentation_1.PDF]

Supplementary method

For production of recombinant enzymes in *E. coli*, the sequences of the modified cytoplasmic α-amylase (amylase$^{\Delta 1\text{-}37}$), based on NCBI sequence Acc. # WP_014835153 (designated as α-amylase in the manuscript) and full length amylopullulanase (Acc.# NC_018092) were amplified from *Pyrococcus furiosus* chromosomal DNA using Phusion Hot Start II High-Fidelity DNA Polymerase (Fisher, Pittsburgh, USA). The sequences were amplified with a pair of oligonucleotide primers 5'-*aaggaaagga*GAATTCatgcaagcattctattgggatgttccagggggag-3' and 5'-*aaagga*GCGGCCGCcttgtcatcatcgtccttgtagtccccaacaccacaataactc-3' for *a*-amylase, and 5'-*aaaggaaagga*_CATATG_agtaggaagctttctctcctcttagtatttct-3' and 5'-*ttcccttcct*_CTCGAG_gctccttcttcttaaaacacctagagctgccaatagtac-3' for amylopullulanase containing restriction enzyme sites (upper case letters and underlined) of *EcoR*I and *Not*I for α-amylase, and *Nde*I (includes the initiation codon 'ATG') and *Xho*I for amylopullulanase. The PCR products were gel-purified using QIAquick Gel Extraction Kit (Qiagen, Valencia, CA), double digested with the respective restriction enzymes and inserted into pET-32a (α-amylase) and pET-24a (amylopullulanase) expression vectors (Novagen, Madison, USA) also digested with the same pair of restriction enzymes, in-frame with N-terminal highly soluble Trx-tag (pET-32a) and C-terminal His-tag (pET-24a) under the control of strong bacteriophage T7 transcription and translation signals. The resulting constructs were fully sequenced for accuracy using gene-specific primers, as well as T7 promoter and terminator primers flanking the multicloning site. The constructs (*pET32-Pf-α-amylase* and *pET24-Pf-pullulanse*) and the empty vectors pET-32a or pET-24a were then introduced into Rosetta 2(DE3)pLysS (*pET32-Pf-α-amylase)* and Bl21 (DE3) (*pET24-Pf-pullulanse*) *E. coli* competent cells (Novagen).

Protein extraction and purification

For recombinant protein production in *E. coli*, 5 mL of LB-media containing appropriate antibiotics was inoculated with a single *E. coli* colony harboring each construct and cultured overnight at 37°C. Then 1 mL of the overnight culture was used to inoculate 100 mL of LB medium containing appropriate antibiotics and incubated at 37°C while shaking until the cell density (OD600) reached between 0.4-0.6. Then 25 mL of the culture was treated with 0.5 mM

for a-amylase, and 1.0 mM isopropyl β-D-1-thiogalactopyranoside (IPTG), and the culture was grown for 6 h (α-amylase) and 12 h (pull). After induction with IPTG, cells were lysed using BugBuster Protein Extraction Reagent (Novagen) containing Benzonase Nuclease to reduce sample viscosity, and Protease Inhibitor Cocktail Set III (without EDTA). The lysate was centrifuged at 16,000 x g for 20 min at 4^oC. Total soluble protein in the supernatant was recovered and heat-purified at 70^oC for 25 min, and centrifuged at 19000 x g for 20 min before it was used for enzyme assay or purified using His-Select Ni-affinity gel (Sigma, St. Louis, USA). Protein concentration was determined using BCA protein assay Kit (Novagen) using bovine serum albumin (BSA) as a standard.

Assay of Enzyme activity

The activity of the hyperthermophilic enzymes was determined using the glucose oxidase-peroxidase method (Sigma) with minor modification. Starch degrading ability of α-amylase was determined in an assay mixture containing 50 mM phosphate buffer (pH7.0), 1% of potato or corn starch (Sigma) in the presence of 50 μl total heat-protein for α-amylase. The activity of amylopullulanase was assayed in Na-acetate buffer (50 mM) containing 0.5 mM $CaCl_2$, 1% pullulan solution as substrate, 100 μl (total) or 25 μl (purified) enzyme and the reaction was incubated at 80^oC for 20 min. The assay reaction contained 500 μl of the hydrolysate and 1 mL of the assay reagent containing glucose oxidase-peroxidase enzymes, and incubated at 37^oC for 30 min. The reaction was stopped by adding 1 mL of 12N H_2SO_4. Increase in absorbance at 540 nm was determined using a spectrophotometer and the amount of released glucose was determined from standard curve of known glucose concentration.
